# Case report of pure single-port robotic left lateral sectionectomy using the da Vinci SP system

**DOI:** 10.1097/MD.0000000000028248

**Published:** 2021-12-23

**Authors:** Wan-Joon Kim, Pyoung-Jae Park, Sae-Byeol Choi, Wan-Bae Kim

**Affiliations:** a Division of Hepatobiliary Pancreas Surgery, Department of Surgery, Korea University Guro Hospital, Korea University Medical College, Seoul, Korea; b Division of Transplantation Vascular Surgery, Department of Surgery, Korea University Guro Hospital, Korea University Medical College, Seoul, Korea.

**Keywords:** case report, da Vinci SP, liver resection, robotic surgery

## Abstract

**Introduction:**

Since its first appearance in the early 1990s, laparoscopic hepatic resection has become increasingly accepted and recognized as safe as laparotomy. The recent introduction of robotic surgery systems has brought new innovations to the field of minimally invasive surgery, such as laparoscopic surgery. The da Vinci line of surgical systems has recently released a true single-port platform called the da Vinci SP system, which has 3 fully wristed and elbowed instruments and a flexible camera in a single 2.5 cm cannula. We present the first case of robotic liver resection using the da Vinci SP system and demonstrate the technical feasibility of this platform.

**Patient concerns and diagnosis:**

A 63-year-old woman presented with elevated liver function test results and abdominal pain. Computed tomography (CT) and magnetic resonance cholangiopancreatography showed multiple intrahepatic duct stones in the left lateral section and distal common bile duct stones near the ampulla of Vater.

**Interventions:**

The docking time was 8 minute. The patient underwent successful da Vinci SP with a total operation time of 135 minute. The estimated blood loss was 50.0 ml. No significant intraoperative events were observed.

**Outcomes:**

The numerical pain intensity score was 3/10 in the immediate postoperative period and 1/10 on postoperative day 2. The patient was discharged on postoperative day 5 after verifying that the CT scan did not show any surgical complications.

**Conclusion:**

We report a technique of left lateral sectionectomy, without the use of an additional port, via the da Vinci SP system. The present case suggests that minor hepatic resection is technically feasible and safe with the new da Vinci SP system in select patients. For the active application of the da Vinci SP system in hepatobiliary surgery, further device development and research are needed.

## 1 Introduction

Since its first appearance in the early 1990s, laparoscopic hepatic resection has become increasingly accepted and recognized to be as safe as laparotomy.^[1–5]^ Initial forays into the field involved laparoscopic fenestration of liver cysts and other cases involving transection of a small amount of liver tissue. Beginning with the publication of a study showing that there were no differences between laparoscopic lateral sectionectomy and open lateral sectionectomy in terms of surgical complications and oncological outcomes, laparoscopic liver resection has been gradually expanding in terms of its range of applications.^[6,7]^ Laparoscopic liver resection benefits patients in terms of better cosmetic outcomes, less pain, and shorter hospital stay due to earlier recovery.^[8–10]^

The recent introduction of robotic surgery systems has brought new innovations to the field of minimally invasive surgery, such as laparoscopic surgery.

It was developed to overcome the disadvantages of conventional laparoscopic surgery. The well-known advantages of robotic surgery, such as improved vision of the surgical field via 3D vision, visual magnification, tremor suppression, and instrument flexibility and dexterity, have allowed precise and meticulous operation techniques.^[11–13]^

In Korea, robotic surgical systems were first adopted in the general surgical field of cholecystectomy in 2005. In 2008, robotic single-site surgery was introduced with a single gel port accommodating multiple trocars for a camera, 2 arms, and an assist port.

Robotic liver resection has been reported to be feasible and safe on the basis of experiences at specialized single centers.^[14,15]^ However, robotic single-port surgery using the da Vinci Si and Xi has limitations in endowrist motion. Furthermore, when assisted surgery procedures, such as traction, are required, assistance may not be available, or an additional assist port may need to be created. This has the effect of offsetting the advantages of robotic surgery. The da Vinci system has recently released a true single-port platform called the da Vinci SP system, which has 3 fully wristed and elbowed instruments and a flexible camera in a single 2.5 cm cannula.

We present the first case of robotic liver resection using the da Vinci SP system and demonstrate the technical feasibility of this platform.

## 2 Procedures

### 2.1 Case presentation

A 63-year-old woman presented with elevated liver function test results and abdominal pain.

The patient had dyslipidemia and was taking hyperlipidemic drugs. Computed tomography (CT) and magnetic resonance cholangiopancreatography showed multiple intrahepatic duct stones in the left lateral section and distal common bile duct stones near the ampulla of Vater. We removed the distal common bile duct stone via endoscopic retrograde cholangiopancreatography (ERCP). We planned to perform robotic left lateral sectionectomy using the da Vinci SP system. Three days after ERCP, clinical and laboratory examinations showed no biliary obstructive symptoms. We performed the operation thereafter. This case report was conducted after obtaining informed consent and acquiring approval from the ethics committee of Korea University Guro Hospital.

### 2.2 Operative procedure

The patient underwent standard bowel preparation and received prophylactic antibiotics.

**Figure 1:**
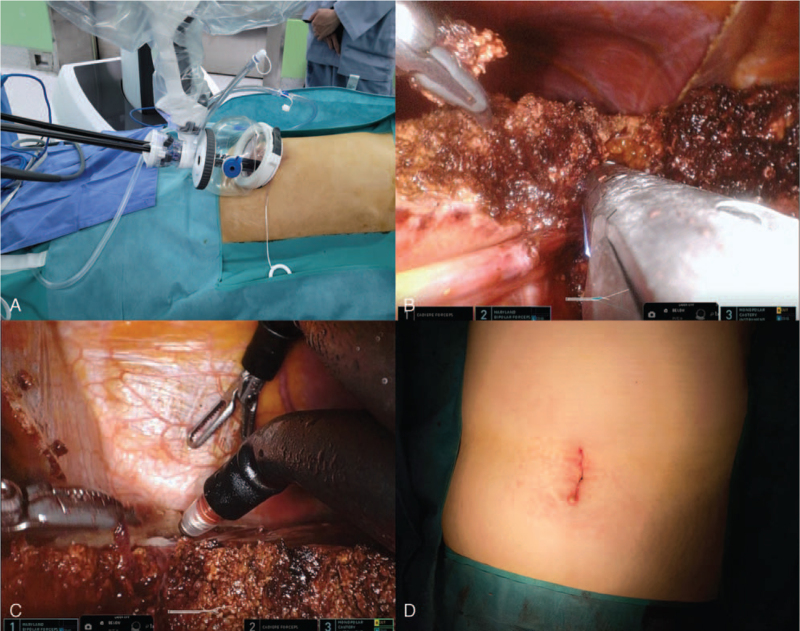
Intraoperative procedure of the robotic SP left lateral sectionectomy. (A) External view of the docked da Vinci SP system. (B) Portal pedicles were divided using an endoscopic linear stapler. (C) View of the dissection near left hepatic vein root using endo-wristed 3 robot arm. (D) 3 cm skin incision at the umbilicus.

A 3 cm vertical incision was made at the umbilicus. A uniport device (Da Vinci SP Access Port Kit, Intuitive Surgical, Sunnyvale, CA) was applied using a suction device and an endosurgical stapler. The da Vinci single 2.5 cm trocar was then inserted and connected to an insufflator. After changing the patient's position to the reverse Trendelenburg with the right side up, the trocar was docked to the da Vinci SP patient side cart arm. The camera was inserted into the lower middle hole. Fenestrated bipolar forceps were placed at the left hole (arm 1), Cadiere forceps were placed at the upper-middle hole (arm 2), and monopolar forceps were placed on the right hole (arm 3). After docking and setting up the instrument, the round ligament was separated from the abdominal wall, and the left triangular ligament was dissected to allow the left liver to move. Superolateral traction of the detached round ligament was accomplished using arm 2. The hepatic parenchyma was divided along the right side of the falciform, and the pedicles to segment IV were divided without using the Pringle maneuver, by using endowristed monopolar forceps (arm 3) and bipolar forceps (arm 1) with the Kelly clamp crushing method. Larger structures were secured using Hem-o-lok clips. The portal pedicles and major hepatic veins were divided using an endoscopic linear stapler (ECHELON FLEX Powered Vascular Stapler; Johnson & Johnson, New Brunswick, NJ). After the resected specimen was completely divided, it was inserted into an endobag. After carefully performing hemostasis, fibrin glue and hemostatic materials were applied to the surface of the dissected liver. After checking for hemostasis and bile leak, instruments were withdrawn, the patient cart arm was unlocked, and the specimen was pulled out with a single-site port and a uniport device (Fig. 1).

### 2.3 Peri- and postoperative outcomes

The docking time was 8 minute. The patient underwent successful operation with the da Vinci SP system, and the total operation time was 135 minute. The estimated blood loss was 50.0 ml. No significant intraoperative events were observed. The numerical pain intensity score was 3/10 in the immediate postoperative period and 1/10 on postoperative day 2. The patient was discharged on postoperative day 5 after verifying that the CT scan did not show any surgical complications.

## 3 Discussion

To date, robotic surgery using a single-port platform has been performed in various procedures, including hysterectomy, prostatectomy, and cholecystectomy.^[16–18]^ To the best of our knowledge, the current report is the first to show the use of the da Vinci SP system in hepatic anatomical resection. Robotic surgical systems have several advantages compared with laparoscopic surgery, and their applications have been gradually expanded to various fields and more complicated procedures.

Giulianotti et al^[19]^ reported an initial experience with robotic liver resection in 2002. Robotic liver resection has been reported to be feasible and safe on the basis of experiences at multiple specialized single centers. However, the limited variety of equipment and the high cost of robotic surgery compared with laparoscopic liver surgery can be obstacles to the adoption of robotic liver surgery by surgeons who are not experienced in robotic surgery. Therefore, the potential advantages of robotic surgery remain controversial.

Wang et al^[20]^ showed that robot-assisted minor hepatectomy was associated with a longer operative time than laparoscopic minor hepatectomy and that the cost of surgery was significantly higher in the robotic hepatectomy group than the laparoscopic hepatectomy group. Only Salloum et al^[21]^ reported that the total costs were similar between the 2 groups in a single-center study. More prospective or multicenter studies are needed to assess the feasibility and operative outcomes. Lai et al^[22]^ analyzed the experience of surgeons in Hong Kong with robotic hepatectomy and indicated that surgeons for robotic liver surgery should have the following qualifications: familiarity with liver anatomy, experience in open liver surgeries and in handling emergency situations, adequate training in laparoscopic surgery, and adequate training in robotic surgery.

The da Vinci SP system enables surgeons to perform delicate and complex operations through 1 small incision. The da Vinci SP system consists of several key components, including an ergonomically designed console where the surgeon sits while operating, a patient-side cart where the patient is positioned during surgery, interactive robotic arms, a 3D HD vision system, and a proprietary endowrist arm. The distal triangulation of the SP robot arm provides a greater degree of freedom of movement, particularly in narrow and deep access areas. However, there are limitations to the SP system. Currently, energy devices and staplers, such as the Cavitron Ultrasonic Surgical Aspirator (CUSA), that are compatible with the SP system have not been developed.

The feasibility of parenchymal transection using a robotic approach is critical because bleeding risk increases during this step, and devices for parenchymal resection are significantly limited in robotic surgery compared with laparoscopic surgery. Given the lack of relevant instruments, such as the CUSA or vessel sealing system, the majority of surgeons utilize a combination technique with the Kelly clamp crushing method for parenchymal transection in robot-assisted liver resection. In addition, it is difficult to efficiently deliver the required materials, such as gauzes, sutures, endo-bags, and suction devices, without making additional ports in the SP system. The authors were able to overcome this limitation to some extent by using a uniport, a stapler device, and a suction device without an additional port. A single-port system with a single insertion site and 1 remote center is known to have advantages in terms of pain relief for patients. Several studies have reported conflicting results regarding the effect of the reduction in the number of ports and remote center on postoperative pain.^[18,23,24]^ Our patient also showed good pain level with an NPIS score of 3 points immediately after surgery and a score of 1 point on the discharge date. The da Vinci SP system enables omnidirectional surgery owing to the 360° rotation capability of all instruments, including the camera, within the distance that the instrument can reach. If these advantages are utilized, surgery can be performed in 2 completely different surgical fields at the same time with just 1 port, and the need to make an additional port can be removed. These advantages can maximize the benefits of minimally invasive surgery.

In conclusion, we report a technique of left lateral sectionectomy, without an additional port, via the da Vinci SP system. This case suggests that minor hepatic resection is technically feasible and safe with the da Vinci SP system in select patients. For the active application of the da Vinci SP system in hepatobiliary surgery, further device development and research are needed.

## Acknowledgments

The authors would like to express their sincere appreciation to Kim Ha-Nuel, who served as a physical assistant in our single-port robotic surgery.

## Author contributions

**Conceptualization:** Wan-Bae Kim, Sae-Byeol Choi.

**Formal analysis:** Sae-Byeol Choi.

**Investigation:** Wan-Joon Kim.

**Supervision:** Wan-Bae Kim.

**Validation:** Wan-Bae Kim, Pyoung-Jae Park.

**Visualization:** Pyoung-Jae Park.

**Writing – original draft:** Wan-Joon Kim, Pyoung-Jae Park.
